# Testosterone correlates with Venezuelan equine encephalitis virus infection in macaques

**DOI:** 10.1186/1743-422X-3-19

**Published:** 2006-03-29

**Authors:** Michael P Muehlenbein, Frank B Cogswell, Mark A James, James Koterski, George V Ludwig

**Affiliations:** 1 Department of Anthropology, University of Wisconsin-Milwaukee, USA; 2 Department of Bacteriology and Parasitology, Tulane National Primate Research Center, Covington, Louisiana, USA; 3 Department of Tropical Medicine, Tulane University School of Public Health and Tropical Medicine, New Orleans, Louisiana, USA; 4 Diagnostic Systems Division, United States Army Medical Research Institute of Infectious Diseases, Ft. Detrick, Maryland, USA

## Abstract

Here we briefly report testosterone and cytokine responses to Venezuelan equine encephalitis virus (VEEV) in macaques which were used as part of a larger study conducted by the Department of Defense to better characterize pathological responses to aerosolized VEEV in non-human primates. Serial samples were collected and analyzed for testosterone and cytokines prior to and during infection in 8 captive male macaques. Infected animals exhibited a febrile response with few significant changes in cytokine levels. Baseline testosterone levels were positively associated with viremia following exposure and were significantly higher than levels obtained during infection. Such findings suggest that disease-induced androgen suppression is a reasonable area for future study. Decreased androgen levels during physiological perturbations may function, in part, to prevent immunosuppression by high testosterone levels and to prevent the use of energetic resources for metabolically-expensive anabolic functions.

## Findings

Venezuelan equine encephalitis virus (VEEV) complex, like the Western and Eastern equine encephalitis virus complexes, is a collection of alphaviruses (single, positive strand RNA) of the family Togaviridae that infect humans, rodents and equines in the Americas, and are transmitted via various arthropods, including mosquitoes such as *Aedes, Culex, and Psorophora *[1]. VEEV is a neurotropic virus in laboratory rodents that induces illness characterized first by replication in the lymphoid tissues and development of a high viremia, and second by invasion of the central nervous system via the olfactory neuroepithelium resulting in encephalitis [2-4]. The murine immune response to VEEV is characterized by both Th-1 and Th-2 cytokine gene expression, and both antibody- and cell-mediated responses may play important roles in protection against VEEV [5,6]. Among human and non-human primates, the virus is much less fatal, usually resulting in fever with viremia and lymphopenia [7]. The specific immune responses to VEEV in humans or non-human primates are not well known.

Here we report preliminary results describing the testosterone and cytokine responses to VEEV in macaques which were used as part of a larger study conducted by the Department of Defense to better characterize pathological responses to aerosolized VEEV in non-human primates. Ample evidence suggests that testosterone is inversely associated with measures of immunity in several species, including primates [8-11]. It was therefore hypothesized that testosterone would be directly associated with VEEV viremia and inversely associated with proinflammatory cytokine levels.

Eleven captive-born, adult (mean age: 9.74 years) male cynomolgus monkeys (*Macaco fasicularis*) were maintained in individual steel cages in a BSL-3 facility at the United States Army Medical Research Institute of Infectious Diseases (USAMRIID), Fort Detrick, Maryland. Animals were maintained on a standard laboratory primate diet, and water was available ad libitum. All animals tested negative for antibodies to Western, Eastern, and Venezuelan Equine Encephalitis viruses before their use in this experiment.

Blood samples were obtained on pre-exposure days -60, -45, and -30. On pre-exposure day -30, each animal was implanted with a Data Sciences International TA11PA-C40 Temperature and Activity Telemetry Transmitter. On day 0 (day of exposure), 8 radomly chosen animals were exposed to a total inhaled dose of 1 × 10^8 ^plaque forming units (PFU) of VEEV, Trinidad Donkey strain. Every 24 hours post-exposure, 2 of the 8 infected animals were randomly chosen to be anesthetized and euthanized by exsanguination. Euthanasia and single interval sampling, rather than multiple bleeds throughout infection, were the requirement of another study by the Department of Defense using these same animals.

For each blood sample, animals were anesthetized with 0.1 mg/kg ketamine/acepromazine maleate solution and a 6 ml blood sample was immediately obtained from the femoral vein using a Vacutainer tube with serum separator. Samples were centrifuged, the serum frozen at - 80°C, and aliquots shipped to Yale University and Tulane University. Samples from the two animals which were exposed to VEEV and sampled/sacrificed 24 hours later (day 1) were not available for analysis in the current study, thus the final size was as follows: 3 baseline samples taken from each of 11 animals (N = 33); 1 sample taken from each of 2 animals 48 hours after exposure (N = 2); 1 sample taken from each of 2 animals 72 hours after exposure (N = 2); 1 sample taken from each of 2 animals 96 hours after exposure (N = 2). Because of ethical concerns and costs associated with the use of non-human primates, and because this project utilized animals from a study in progress by the Department of Defense, sample sizes could not be increased for purposes described here.

Research was conducted in compliance with the Animal Welfare Act and other Federal statutes and regulations relating to animals and experiments involving animals and adheres to principles stated in the Guide for the Care and Use of Laboratory Animals, National Research Council, 1996. The facility where this research was conducted is fully accredited by the Association for Assessment and Accreditation of Laboratory Animal Care International.

At Yale University, samples were assayed for total testosterone and cortisol levels using coated-tube radioimmunoassay kits (DSL-4000 and DSL-2100) from Diagnostic Systems Laboratories (DSL), Webster, Texas. The sensitivities of the assays were 0.08 ng/ml for testosterone and 0.3 ng/dl for cortisol. The correlation coefficients for each of the curves were better than 0.99. High and low level DSL controls were included in each standard curve; results for the controls in each assay were within established confidence limits. Intra-assay coefficients of variation were assessed using the mean coefficients of variation of control duplicates. Intra-assay coefficients of variation were 5.8% for testosterone and 9.1% for cortisol. All samples were run in a single assay, therefore there were no inter-assay coefficients of variation to assess. For each individual animal, hormone values from the pre-exposure samples on days -60, -45, and -30 were averaged to yield a baseline (pre-exposure) level.

At Tulane University, samples were assayed for the following cytokines using enzyme-linked immunosorbent assay: IL-1β, IL-4, IL-10, IL-12, TNFα, and IFNγ (Biosource International Inc., Camarillo, CA, USA). Of each of these kits, all but IL-1β (human IL-lβ kit) was designed specifically for use in macaques. The human IL-1β kit demonstrates 100% cross-reactivity with non-human primate samples (S. Durham, BioSource International, Technical Services, personal communication). The sensitivities of the assays were: IFNγ : <4 pg/ml; TNFα : <2 pg/ml; IL-1β : 1 pg/ml; IL-4: <3 pg/ml; IL-10: <10 pg/ml; IL-12: <4 pg/ml. The correlation coefficients for each of the curves were better than 0.96. Intra-assay coefficients of variation were assessed using the mean coefficients of variation of control duplicates. Intra-assay coefficients of variation were: IFNγ: 5.1%; TNFα : 3.7%; IL-1β : 4.7%; IL-4: 5.4%; IL-10: 5.1%; IL-12: 4.3%. All samples were run on a single assay, and therefore there were no inter-assay coefficients of variation to assess.

At USAMRIID, viremia was determined by titration of samples on tissue culture (VERO 76) cells and enumeration of plaques (plaque forming units: PFUs). Viremia was measured only on the day of sacrifice, and therefore this measure does not reflect peak viremia titers.

Data were analyzed using SAS/STAT software (SAS Institute Inc., Gary, NC). The Wilcoxon Signed Rank test was used to test whether the median change in temperature, testosterone, cortisol, or cytokine values from the pre-exposure samples to the sacrifice (exposure) samples were significantly different from zero (no change). Spearman correlations were used to determine associations between the various variables. Cortisol levels were determined and controlled for in (as a covariate) all analyses involving testosterone because cortisol exhibits mainly inhibitory effects on immune function and because cortisol is often associated with suppression of the hypothalamic-pituitary-testicular axis [12,13]. As the immunosuppressive actions of glucocorticoids are better characterized than that of androgens, the focus of this study was testosterone rather than cortisol-related effects. Additionally, level of significance (alpha) was set at 0.10 so as to err on the side of conservatism (to avoid false negatives) given the small sample size. Power analyses for the Wilcoxon Signed Rank tests were performed with the SAS/STAT Analyst Sample Size application for paired t-tests (SAS Institute Inc., Gary, NC) to assess the likelihood of false negatives. Power (%) is reported below for those tests that revealed non-significant results.

Temperature, virus titer, testosterone and cytokine levels were compared between samples taken prior to exposure and on the day of sacrifice (48, 72, or 96 hours post-exposure, depending on animal). Mean temperature at time of sacrifice (37.25°C) was significantly higher (T = 10.5, p = 0.03) than mean temperature prior to VEEV exposure (35.89°C). Mean testosterone level at time of sacrifice (13.37 ng/ml) was significantly lower (T = -8.5, p = 0.094) than testosterone level prior to VEEV exposure (19.51 ng/ml). Table 1 presents viremia and pre-and post-infection testosterone levels for each of the six animals infected with VEEV.

**Table 1:** Viremia and pre- and post-infection testosterone levels for each of six animals infected with VEEV

Animal ID	Viremia (PFU) at time of sacrifice	Time (hrs) between exposure and sacrifice	Testosterone (ng/ml) at time of sacrifice	Pre-exposure testosterone (ng/ml) (mean for each animal)
1	7800	48	15.58	28.14
2	7800	48	20.43	26.29
3	370	72	22.06	16.44
4	370	72	2.81	13.11
5	50	96	12.56	25.07
6	25	96	6.79	8.01

Statistical analyses could only be performed on IL-12 and IL-10 levels because TNFα and IL-4 levels were not above a detectable level for any sample of any animal, and only one animal had IL-1β and IFNγ values greater than zero. Mean IL-12 prior to exposure was 537.36 pg/ml, which was significantly higher (T = -10.5, p = 0.03) than mean IL-12 at time of sacrifice (280.66 pg/ml). Mean IL-10 prior to exposure was 286.35 pg/ml, which was not significantly different (T = -3.0, p = 0.25, power = 20%) from mean IL-10 at time of sacrifice (236.07 pg/ml).

Viremia was available from only 6 of the 8 animals which were exposed to VEEV. Virus titers were highest in animals sacrificed at 48 hours than in those other animals of known viremia. Virus titers for the six animals were as follows: 7800 PFU per ml of blood for the two animals sacrificed 48 hours post-exposure; 370 PFU per ml for the two animals sacrificed 72 hours post-exposure; and 50 and 25 PFU per ml for the two animals sacrificed 96 hours post-exposure.

Pre-exposure testosterone and cortisol levels were both positively associated with viremia levels after exposure (r = 0.98, p = 0.02 and r = 0.96, p = 0.04, respectively; controlling for the hours between exposure and sacrifice; testosterone correlation controlling for cortisol levels, and vice versa).

The results of this preliminary study suggest that: 1) macaques infected with VEEV exhibit a febrile response with possibly few significant changes in cytokine levels; 2) higher levels of testosterone (when controlling for cortisol) prior to virus exposure may be directly associated with higher viremia after exposure; and 3) testosterone levels are lower during infection with VEEV than prior to infection.

First, macaques infected with VEEV exhibited a significant febrile response with few significant changes in cytokine levels. That is, temperature levels were elevated, IL-12 levels were lower, and IL-10 levels were relatively unchanged during infection. In both humans and non-human primates, VEEV infection has been characterized by fever with viremia and lymphopenia [7] Unfortunately, no definitive conclusion can be drawn regarding the cytokine response to VEEV in the present study utilizing macaques because of the small sample size, lack of statistical power, and inability to assess the acute phase of infection (the sample was lacking information from animals at the 24 hour post-exposure timepoint). Furthermore, exposure to VEEV was not followed-up for longer than 96 hours, and only a single sample was obtained from each animal throughout this short infection period.

Second, higher levels of testosterone prior to virus exposure were directly associated with higher viremia after exposure. That is, when controlling for time between exposure and sacrifice as well as cortisol levels, pre-exposure testosterone levels were significantly (directly) associated with viremia in post-exposure samples. In general, the effects of testosterone on viral infections, incluyding HIV and Sindbis virus, are equivocal [11,14,15]. It may be the case that testosterone plays an immunosuppressive role in response to viral infection in male macaques and that higher levels of basal testosterone levels may increase an animal's susceptibility to viral infection. Future studies are warranted.

Thirdly, testosterone levels were significantly lower during infection with VEEV than prior to infection. Similarly, serum testosterone decreases during the onset of various conditions, including *Plasmodium vivax *infection in Honduran men [16], surgery and trauma in men [17], and *Trypanosoma brucei brucei *infection in rats [18]. Furthermore, testicular atrophy and azoospermia have been reported from men who died of AIDS [19], and azoospermia has been associated with SIV infection in young male rhesus macaques [20]. Depressed androgen levels during physiological perturbations may be an advantageous, adaptive host response in order to prevent immunosuppression by high testosterone levels and to re-direct energetic resources [21-23], specifically away from metabolically-expensive anabolic functions [11]. Although the results of the present study are very preliminary, they do suggest that disease-induced androgen suppression is a reasonable area for future study.

## Abbreviations

Venezuelan equine encephalitis virus (VEEV), United States Army Medical Research Institute of Infectious Diseases (USAMRIID), plaque forming units (PFU), human immunodeficiency virus (HIV), acquired immunodeficiency syndrome (AIDS), simian immunodeficiency virus (SIV).

## Competing interests

The author(s) declare that they have no competing interests.

## Authors' contributions

MPM conducted the hormone analyses and wrote the manuscript. FBC edited the paper and coordinated the research efforts at Tulane Primate Center. MAJ edited the paper and conducted the cytokine analyses. JK conducted the animal work. GVL edited the paper and coordinated the research efforts at USAMRIID. All co-authors read and approved the final manuscript.
